# Systemic and mucosal immunity to enterotoxigenic *Escherichia coli*: evidence from natural infection and vaccination

**DOI:** 10.3389/fimmu.2026.1865150

**Published:** 2026-07-16

**Authors:** Cynthia Mubanga, Masauso Moses Phiri, Kapambwe Mwape, Dhvani Kuntwala, Kachana Chaloba, Mutale Mubanga, Mofya Phiri, Caroline Chisenga, Roma Chilengi, Richard H. Glashoff

**Affiliations:** 1 Basic Science and Immunology Unit, Center for Infectious Disease Research in Zambia, Lusaka, Zambia; 2 Division of Medical Microbiology & Immunology, Department of Pathology, Stellenbosch University & National Health Laboratory Service, Tygerberg Hospital, Cape Town, South Africa; 3 Department of Pathology and Microbiology, University of Zambia, School of Medicine, Lusaka, Zambia; 4 Department of Computer Studies, School of Natural Sciences, University of Zambia, Lusaka, Zambia; 5 Zambia National Public Health Institute, Lusaka, Zambia

**Keywords:** ETEC, immune correlates, mucosal immunity, systemic immunity, vaccine development

## Abstract

Enterotoxigenic *Escherichia coli* (ETEC) is a major contributor to diarrheal morbidity and mortality, especially among children under 5 years of age in low- and middle-income countries (LMICs). Development of an effective ETEC vaccine has been impeded by the pathogen’s antigenic complexity and lack of well-defined immune correlates of protection. This review synthesizes evidence from human infection and vaccination studies to summarize current knowledge on systemic and mucosal immune responses to ETEC, the assays employed to measure them, and the factors affecting immune outcomes, providing a critical synthesis of systemic and mucosal immune measures. Most studies reviewed assessed systemic immune responses despite ETEC being an enteric pathogen, highlighting challenges in measuring mucosal immune responses. Substantial progress has been made in assay technologies, from basic singleplex Enzyme-Linked Immunosorbent Assays (ELISAs) to robust multiplex and microarray platforms that enable profiling of responses to multiple antigens and immunogenic antigen discovery. The evidence shows that multiple immune pathways, including humoral, cellular, and mucosal components, contribute to overall protection. The review further highlights the need for assay standardization to facilitate cross-study comparisons and uniform interpretation of immunogenicity and efficacy data. ETEC immunity is complex and is impacted by host, pathogen, and environmental factors. Standardizing immunological assays and defining measurable correlates of protection is critical for the accurate evaluation and optimization of ETEC vaccine candidates.

## 1 Introduction

Diarrhea remains a leading cause of morbidity and mortality, accounting for an estimated 1.17 million deaths globally in 2021 (1). The highest burden is among children under 5, with most cases occurring in Sub-Saharan Africa and South Asia (1–3). Enterotoxigenic *Escherichia coli* (ETEC) is among the leading causes of moderate-to-severe diarrhea (MSD), especially among children and travelers to endemic countries (4–8). The highest burden is seen in children below 2 years who experience recurrent diarrhoeal episodes, which may result in long-term health consequences such as malnutrition, stunting, and impaired cognitive development (1, 9–11).

While improving water quality, sanitation, and hygiene (WASH) is critical for reducing diarrhoeal morbidity, its implementation can be challenging in low- and middle-income countries (LMICs) due to high costs (7). Vaccines, therefore, provide a cost-effective alternative for the prevention and control of infectious diseases as they not only prevent disease and death but also minimize the risk of long-term sequelae such as environmental enteric dysfunction (EED) and antimicrobial resistance (AMR) (7, 12).

Progress in the development of an effective ETEC vaccine has been slow due to several challenges, such as the pathogen’s extensive antigenic diversity, the lack of well-defined immunological correlates of protection, and the complexity of eliciting both systemic and mucosal immune responses. Added to this is the challenge in accurately measuring mucosal responses (7, 13–15).

ETEC strains exhibit extensive heterogeneity, particularly in colonization factors (CFs), with more than 25 identified, while toxin diversity is relatively limited and comprises the heat-labile (LT) and heat-stable (ST) enterotoxins. These features require broad immunogenic coverage to ensure protection (13, 16). Consequently, vaccine development has largely focused on specific CFs, toxins, and other defined virulence-associated proteins rather than conserved non-virulence “housekeeping” antigens (17, 18). The most advanced candidate vaccines (ETVAX and ACE527) are therefore designed to express the epidemiologically prevalent CFs and toxin component to induce a broadly protective immune response (7).

Understanding the immunological responses to ETEC, following both natural infection and vaccination, is important for informing optimal vaccine design and development (9, 13). Immunological assessment primarily involves measuring systemic immune markers such as serum immunoglobulins (Ig) A, G, and M, circulating memory B cells, and T-cell activation. This is despite the fact that mucosal immunity, particularly secretory IgA and local tissue-resident immune populations, plays a central role in protection against enteric pathogens (13, 19). Moreover, the nature, magnitude, and durability of these responses can differ by age, geographic location, prior exposure, and the immunization platform used (13).

This narrative review seeks to summarise current knowledge on systemic and mucosal immune responses to ETEC, drawing on findings from natural infection studies, human challenge models, and vaccine trials. It highlights key immunological themes, assay methodologies, sample types, and age-specific or geographic patterns with the goal of identifying gaps in the current status of the field and ongoing efforts to advance understanding of ETEC vaccine development and immunogenicity.

## 2 Approach

For this comprehensive review of systemic and mucosal immune responses to ETEC, we searched several databases, including Medline via PubMed, Web of Science, Scopus, Trip, Cochrane, World Health Organization (WHO) global health library, and ClinicalTrials.gov registry, and synthesized the relevant findings. We used combinations of the terms “ETEC,” “enterotoxigenic Escherichia coli,” “immune response,” “vaccine,” “natural infection,” “challenge studies,” and “mucosal immunity” to identify suitable articles. All electronically available peer-reviewed articles or conference papers of original studies with data on the immune responses to ETEC in both adults and children published in English were included. The studies of interest were either vaccine trials assessing immunogenicity, human infection challenge studies, or studies on ETEC natural infection reporting on numbers of immune cells [T-cells, B-cells, and antibody-secreting cells (ASCs)], the changes in ETEC-specific antibody (IgA and IgG) titers, and cytokine production by T-cells.

Thematic synthesis was conducted for group studies by type of immune response (systemic vs. mucosal), nature of exposure (natural infection, vaccination, or challenge), population (e.g., age group, geographical setting), and methods used for immune marker assessment.

## 3 ETEC pathogenesis

ETEC is an enteric pathogen that causes disease through non-invasive mechanisms. According to the classical paradigm of pathogenesis, ETEC attaches to enterocytes via fimbriae-like structures known as colonization factor antigens (CFAs) or colicin surface antigens (CSs) following ingestion (20–23). After attachment, the bacteria release enterotoxins LT or ST that enter the cell and activate intracellular adenylate cyclase (by LT) or guanylate cyclase (by STa), causing a disruption of homeostasis in the host cell through alteration of ion channels, leading to the flow of water and salt into the intestinal lumen, resulting in watery diarrhea (20, 22).

In recent years, several other proteins and virulence factors have been identified. These non-classical ETEC antigens, such as the serine protease EatA and the adhesin EtpA, together with conserved factors like type 1 fimbriae, EaeH, surface autotransporter proteins, and the YghJ metalloprotease, help the bacteria attach to the intestine and deliver their toxins more effectively (13, 21, 23–25). These antigens appear to be relatively conserved across ETEC strains and have been shown to induce antibody responses in human challenge studies and proteomic analyses, supporting their potential contribution to protective immunity (13, 20, 25).

Unlike invasive enteropathogens, protection against ETEC is thought to rely predominantly on mucosal immune responses that prevent intestinal colonization and neutralize enterotoxins.

Protective antigens are generally considered to be those capable of eliciting immune responses that confer protection against infection or disease (13, 26). Consequently, vaccine design has focused on selecting antigens based on their roles in pathogenesis, epidemiological prevalence, degree of conservation, and ability to elicit functional immune responses associated with protection, with the goal of achieving broad strain coverage (13, 23).

## 4 Immune responses to ETEC exposure

### 4.1 Systemic immune responses

#### 4.1.1 Humoral (serum IgA, IgG, IgM, ASCs, ALS)

The humoral immune response, mediated through the production of antibodies, plays a central role in host defence against extracellular pathogens such as ETEC.

Complementing mucosal immune responses, systemic humoral immunity involves antibodies that circulate in the bloodstream and contribute to host defence (27). These are mainly immunoglobulins IgA, IgG, and IgM, typically measured by determining their relative concentrations in serum or plasma, or by antibody secretion assays using peripheral mononuclear cell (PBMC)- derived antibody. The antibodies target various ETEC virulence factors, including LT, CFs, lipopolysaccharide (LPS), and several non-canonical antigens such as EatA, EtpA, and YghJ (20, 25, 28). While these responses may sometimes be transient with differing kinetics for the different antibody isotypes, they provide insights into elements of immune priming following infection, challenge or vaccination. However, the extent to which circulating antibody responses accurately reflect protective mucosal immunity remains incompletely understood.

##### 4.1.1.1 IgA responses

Systemic IgA responses rise rapidly, within 14 days following both vaccination and natural infection, typically peaking around day 7 after ETEC exposure (29–33). The magnitude and persistence of these responses vary according to the antigen, the immune compartment measured, and the type of exposure (vaccination or natural infection). In natural infection, IgA levels measured in both serum and antibody in lymphocyte supernatant (ALS), which reflects antibodies actively secreted *in vitro* by short-lived plasmablasts from blood, peaked by day 7 and declined by day 21 in both adults and children (30). Differences in IgA responses have also been observed between natural infection and vaccination. Wennerås et al. found stronger IgA ASC responses after natural infection than after vaccination with a killed whole-cell ETEC vaccine containing recombinant CTB and the major colonization factors CFA/I and CFA/II, with IgA predominating over the IgG and IgM isotypes in the blood (34).

Circulating ASC responses peak early and decline by approximately day 30, whereas memory B-cell responses and antibody avidity may persist beyond this period, suggesting continued maturation of humoral immunity (32, 33). Furthermore, responses differ between antigens, with LTB-, CFA/I-, and CS6-specific responses exhibiting distinct kinetics and patterns of cross-reactivity (33). After ETEC infection, there is a brief, predominantly gut-directed, IgA-dominant immune response that peaks around day 7 and then declines. These observations suggest that blood IgA measurements may provide an indirect indication of ongoing mucosal immune responses.

Patients have been reported to have significant increases in total IgA- and IgG-secreting cells expressing the gut-homing integrin α4β7 on day 7 following infection, with numbers declining by day 30 (32). These β7+ cells, primarily expressing the α4β7 heterodimer, facilitate mucosal homing via interaction with mucosal vascular addressin cell adhesion molecule-1 (MAdCAM-1). Importantly, most IgA detected in circulation is monomeric, whereas secretory IgA at mucosal surfaces is predominantly dimeric and is transported across the intestinal epithelium via the polymeric immunoglobulin receptor (pIgR) (28). Consequently, circulating IgA measurements provide only an indirect surrogate of protective mucosal immunity and should therefore be interpreted with caution when inferring intestinal immune protection (28, 30, 34).

Serum IgA responses after ETEC infection may contribute to protective immunity. Early studies reported robust antitoxin responses in hospitalized patients with ETEC diarrhea, with 60–75% demonstrating LT-neutralizing activity (35). Collectively, these findings suggest that although IgA responses are often transient and exhibit antigen-specific kinetics, they represent an important component of protective immunity following ETEC exposure. However, the extent to which circulating IgA responses accurately reflect protective mucosal immunity remains incompletely understood.

##### 4.1.1.2 IgG responses

Systemic IgG responses also rise rapidly after exposure but tend to be more sustained than IgA.

Both gut-destined β7+ IgG-secreting cells and plasma IgG responses to CS6 and LTB have been reported to peak on day 7 and remain elevated until day 30 (32, 33). In a volunteer challenge study, Evans et al. reported that 12 of 13 volunteers infected with a CFA/I-expressing ETEC strain (E. coli H-10407) showed more than a fourfold increase in serum IgG titers to LT, CFA/I, and the bacterial O antigen (36).

In a study examining rates of LT-specific IgG seroconversion among US travelers to Mexico, seroconversion was observed in 74.1% of symptomatic travelers compared to 22% of asymptomatic travelers (37). Similarly, IgG titers were shown to correlate with IgA ASC responses in African participants receiving a toxoid oral, killed ETEC plus cholera toxin B subunit vaccine (38).

The presence of systemic IgG to a particular antigen may serve as a useful biomarker of previous exposure to ETEC antigens. For example, a study showed that US students exposed to ETEC during travel developed marked IgG responses to somatic O and colonization antigens (39). Consistent with these findings, in our previous work measuring IgG responses in children participating in an ETVAX^®^ vaccine trial, we observed plasma IgG responses to both canonical and non-canonical ETEC antigens prior to vaccination, suggesting previous exposure to ETEC.n (25). While the magnitude and kinetics of IgG responses are well described, their functional roles are not extensively investigated. A few studies report toxin neutralization activity, particularly against LT (35, 40, 41), but the fragment crystallizable region (Fc) -mediated functions of IgG, such as opsonization or complement activation, are not well characterized and warrant further investigation. Thus, although IgG responses are readily induced and relatively durable, their contribution to protection against ETEC beyond toxin neutralization remains incompletely understood.

##### 4.1.1.3 IgM responses

In contrast to IgA and IgG, IgM responses are less frequently measured but serve as early indicators of primary immune activation. IgM responses have been reported against LT, CFs, and LPS, with responses typically peaking early and declining rapidly, as expected when class switching occurs (42).

While less prominent in long-term immunity, IgM detection may serve as an indicator of recent pathogen exposure or initial vaccine priming. Brussow et al. observed that the prevalence of IgM increased with the age of the children from 12% in the first year to 63% by the fourth year, indicative of infections over time (43, 44). This finding of age-related increases in IgM echoes that of an earlier study by Evans et al. who reported acute phase IgM responses mainly among younger participants in controlled challenge settings (36).

Consistent with its role in the primary immune response, serum CFA/II-specific IgM increased one to two weeks following experimental challenge, whereas IgA and IgG responses remained largely unchanged over the same period (45). Collectively, these findings suggest that although IgM responses are transient and contribute little to long-term immunity, they provide useful markers of recent exposure and primary immune activation.

##### 4.1.1.4 ALS and ASC

ASCs and ALS represent systemic proxies for mucosal antibody priming. These are derived from PBMCs following infection or vaccination and are frequently used to quantify antigen-specific IgA and IgG. While ASC assays enumerate antigen-specific antibody-secreting cells directly, ALS assays measure antibodies secreted into culture supernatants by activated plasmablasts, and both have been widely used as indirect indicators of mucosal immune responses (46).

Åhrén et al. measured ALS and ASC responses to various CF antigens and the B subunit of the cholera toxin (CTB), noting robust peaks 7–9 days post-primary vaccination and as early as 4–5 days post-booster (43, 44). Lundgren et al. also observed that IgA ALS responses peaked on day 4–5 following a single dose in previously immunized individuals (47, 48). Notably, ASC kinetics mirror systemic IgA trajectories and have been used to infer immune memory (49).

Wennerås et al. demonstrated higher ASC responses in natural infection compared to vaccination, with IgA remaining the dominant isotype, underscoring the immunological relevance of ALS and ASC assays in both exposure contexts (34).

##### 4.1.1.5 Summary and observations

In summary, systemic antibody responses to ETEC infection or vaccination typically peak around day 7, with IgA showing rapid but transient kinetics, and IgG demonstrating greater durability. While IgM is infrequently assessed, its presence may reflect recent exposure. Surrogate markers of mucosal immunity, such as ALS and ASCs, are measured through ex-vivo assays using PBMCs and provide a less invasive and less labor-intensive means of assessing mucosal immune priming compared to direct sampling of the intestinal mucosa (e.g. intestinal biopsies, lavage or gut aspirates). These approaches are more feasible for use in both clinical and field settings and have shown correlations with serum antibody responses.

Despite being indirect indicators of mucosal immunity, systemic humoral measures remain the most widely reported and standardized readouts across ETEC studies, underpinning their central role in vaccine evaluation and immunological surveillance. However, the extent to which systemic antibody responses accurately reflect protective mucosal immunity remains incompletely understood.

#### 4.1.2 Systemic cellular immune responses

While humoral responses are generally regarded as primary for protection against ETEC, systemic cellular responses are critical in determining the quality of the antibody response. In addition, cellular immune responses contribute to the development of immunological memory and may play important roles in long term protection. Various studies have investigated the role of different cellular immune components in the context of ETEC natural infection, vaccination, and controlled human challenge studies.

##### 4.1.2.1 T cell responses and cytokine production

Early vaccine studies provided initial evidence that oral immunization against ETEC elicit circulating T-cell responses. *In vitro* stimulation with CFA/I and CFA/II induced modest proliferative responses largely by CD4+ T cells (and to a lesser extent CD8+ T cells) with interferon gamma (IFN-γ) production, while interleukin (IL-2) was not detectable (50). These findings point to an important role for CD4 T cells in protective immune responses against ETEC.

Subsequent studies have delved deeper into systemic cellular responses, characterizing the cell subsets involved and their associated cytokine profiles. Analysis of PBMCs from individuals vaccinated with ETVAX^®^ and the dmLT adjuvant collected at baseline and 7 days after the second dose, revealed predominant Th17 and Th1 type T cell responses in the form of increased production of IL-17A and IFN-γ post vaccination (51) These findings suggest that ETEC vaccination induces mixed cellular responses that engage both inflammatory and mucosal pathways. This is may be possible as Th17 responses are known to enhance mucosal defences, and Th1 responses are the primary driver of macrophage activation for elimination of phagocytosed bacteria and their products (52–55).

The role of the double mutant heat-labile toxin (dmLT) adjuvant in shaping these responses has also been highlighted. Bernstein et al. demonstrated that the dmLT adjuvant enhanced cytokine production, including IL-1β, IL-6, IL-23, and IL-12p70, thereby promoting IL-17A responses with downstream effects on mucosal IgA production (56). Similarly, Akhtar et al. showed that dmLT boosts the production of various cytokines such as IL-1β, IL-17A, IL-6, IL-23, IL-4, and IL-12p70 both in infants and adults (57) These enhanced IL-17 responses were associated with enhanced mucosal IgA responses (57).

More recently, Akhtar et al. demonstrated that natural ETEC infection in adults induced predominantly Th17-type responses, with IL-17A responses directed against LT and CS6 antigens and detectable IFN-γ responses in some individuals (58). IL-17A and IFN-γ responses correlated with intestine-derived plasmablast responses, suggesting that T helper responses may contribute to the development of protective IgA responses These findings reinforce earlier observations that Th17-associated responses represent an important component of vaccine- and infection-induced immunity.

Evidence from human challenge studies further supports a possible role for cellular responses in modulating disease outcome.

In volunteers challenged with the CFA/I-positive ETEC strain H10407 (O78:H11, LT+, ST+, CFA/I+), those who did not develop moderate-to-severe diarrhoea showed significantly higher PBMC production of TNF-α, IL-2, IFN-γ, and IL-17A on day 3 after infection, together with increased CD154 (CD40L) expression (59). However, these findings should be interpreted cautiously. The absence of diarrhoea in these individuals does not by itself establish immune-mediated protection, as reduced susceptibility may also reflect limited intestinal colonization or host factors affecting bacterial attachment, including variation in epithelial receptor availability. Thus, the observed cytokine responses are best regarded as correlates associated with reduced disease severity, potentially acting alongside differences in host susceptibility and colonization efficiency, rather than definitive evidence of protection mediated solely by cellular immunity.

Additional support for the importance of pro-inflammatory cellular responses comes from studies in children with ETEC infection. Long et al. showed that zinc-supplemented children with ETEC infection had elevated levels of TNF-α and IFN-γ in PBMCs following ex vivo stimulation with ETEC antigens (60), further underscoring the contribution of cellular immune responses to host defence.

##### 4.1.2.2 Peripheral follicular helper T cells

Peripheral T follicular helper (pTfh) cells have been recognised as facilitators of antigen-specific IgA memory development. McArthur et al. showed that volunteers challenged with the CFA/I-positive ETEC strain H10407 had a lower likelihood of developing diarrhoea when they had a higher proportion of α4β7-positive pTfh cells co-expressing CXCR3 and CCR6 (59). These pTfh populations correlated with stronger IgA responses to ETEC antigens (59). In addition, resistant volunteers exhibited higher ETEC LPS-specific memory B-cell responses 14–28 days following challenge, supporting a link between pTfh responses and the development of humoral memory (59).

Cardeno et al. extended these findings in a vaccine context and observed significant increases in the proportion of circulating Tfh-like CD4^+^CXCR5^+^ T (cTfh) cells expressing activation marker inducible co-stimulator (ICOS) in individuals classified as ASC responders (61). These cTfh cells also showed enhanced expression of IL-21, Th17 markers, and integrin β7, indicative of functional capacity and gut-homing capacity (61). Notably, the magnitude of cTfh responses measured within 1 week of immunization predicted mucosal IgA memory 1–2 years later, following a late booster dose (61). These findings highlight the potential role of pTfh cells in coordinating B-cell responses and generating durable mucosal immune memory.

Although studies investigating cellular immunity to ETEC remain relatively limited compared with the extensive literature on humoral responses, recent advances have enabled more integrated analyses of vaccine- and infection-induced cellular responses. For example, Rim et al. employed mass cytometry to characterize lymphocyte dynamics following experimental human ETEC infection, providing a high-dimensional view of cellular responses (62). Together with multiplex cytokine profiling and proteomic microarrays, these approaches may facilitate identification of composite correlates of protection that are not apparent from individual biomarkers alone. However, systems-level characterization of ETEC immunity remains in its infancy. In particular, systems serology approaches evaluating Fc-mediated antibody functions and multidimensional antibody signatures have not been widely applied in human ETEC studies and represent an important area for future investigation.

#### 4.1.3 Innate systemic responses

Systemic innate immune responses in the context of ETEC infection have been infrequently characterized. In a study by Sheik et al., children with ETEC diarrhea exhibited reduced complement component C3 levels, decreased phagocytic activity in monocytes and neutrophils, increased oxidative burst, and increased peripheral leukocytes and PMNs during acute infection (63). These changes occurred despite ETEC being a non-invasive pathogen, suggesting systemic dysregulation potentially linked to mucosal immune activation (63). Such responses may reflect immune activation secondary to intestinal inflammation and disruption of mucosal homeostasis, or direct effects of enterotoxins on innate immune cells. Indeed, recent studies have shown that, unlike the heat-stable toxin pSTa, heat-labile toxin (LT) directly modulates neutrophil migration, phagocytosis, inflammatory mediator production, and neutrophil extracellular trap formation through cAMP/PKA and ERK1/2 signaling pathways, suggesting a potential mechanism by which ETEC may subvert innate immune responses and facilitate infection (64).

### 4.2 Mucosal immune responses

Mucosal immune responses play an important role in defence against enteric pathogens such as ETEC primarily through the production of secretory IgA (SIgA) at the intestinal mucosal surface which prevents bacterial adhesion and neutralizes toxins (27). These responses target various ETEC antigens and have been measured in stool, gut lavage, intestinal secretions, duodenal biopsies, and breast milk. Other immune factors, such as antimicrobial peptides (AMPs), an important component of mucosal innate immune function, also contribute to the protection of the gut against infection (65).

#### 4.2.1 Humoral responses (stool IgA, mucosal IgG, breast milk)

Evidence from natural infection shows that secretory IgA (SIgA) is produced in the acute phase of infection and can be detected in fecal samples (66). Qadri et al. reported that antibodies were detected in stool on day 7 of infection and response declined by day 21 (30). Estrada-Garcia et al. further observed that in the convalescent phase, 86% of patients had SIgA antibodies to their homologous ETEC strains (66). Together, these findings support the view that mucosal antibody responses arise rapidly following infection and remain detectable into recovery.

Similar patterns have been described following vaccination. Wenneras et al. observed that oral vaccination induced significant IgA ASC responses in duodenal tissue 7 days post vaccination (34). These duodenal ASC responses in the vaccinees were comparable to those observed following natural infection and were predominantly IgA and IgM, while IgG levels rose later during infection (34). High baseline mucosal responses were observed in this cohort and attributed to prior exposure in endemic settings (34).

Differences in the measurement of antibodies across sample types have also been observed. Ahren et al. reported that two doses of an oral vaccine induced significant CFA-specific IgA titres in 61 to 87% of vaccinees when measured in lavage fluid and 38 to 81% when measured in stool (43). The strongest responses were detected against CFA/I, showing that responses to different antigens vary. Furthermore, the levels of mucosal antibodies against CTB and CFA/I in stool correlated significantly with those in intestinal lavage (43). They noted that although stool samples showed good responses, especially against CTB, they were not as sensitive for all antigens compared to lavage (43). Additional support for fecal sampling comes from Ouwehand et al. who observed vaccination-induced increases in LTB-specific SIgA titers in fecal extracts (45). In the same study, fecal calprotectin (a marker of mucosal inflammation) and total IgA peaked on day 2 and 3, respectively, before returning to pre-challenge levels on day 15 (45).

Broader evidence from natural infection shows that mucosal antibody responses can be detected across multiple compartments.

Stoll et al. measured responses to LT, LPS and CFA/I in saliva, serum, breast milk and intestinal lavage during natural infection in an endemic setting (27). They found greater than 2-fold rise in local IgA levels in all sample types, although the magnitude of responses varied among patients (27). Peak IgA responses were recorded on day 9 and declined by day 28, while IgG remained elevated throughout the study period (27). Notably, no LPS responses were detected in saliva (27) Consistent with these findings, although based on a small cohort, El-Mohamad showed that fecal secretory IgA increased more than 2-fold during the acute phase of illness (67). This observation, though based on a small sample size, further supports the critical role of SIgA in the initial stages of infection, to prevent pathogen attachment to the epithelial surface.

Intestinal mucosal responses can be enhanced using adjuvants.

Svennerholm et al. observed that dmLT adjuvant significantly improved intestinal anti-O78 LPS responses in children (68). Similarly, Qadri et al. also noted that adjuvant dmLT improved magnitude, breadth and kinetics of vaccine responses in infants. Interestingly, they observed that dmLT enhanced fecal but not ALS responses (69).

#### 4.2.2 Cellular responses

Cellular mucosal responses involve several immune cell populations, including mucosal T cells, intraepithelial lymphocytes (IELs), and regulatory T cells (Tregs). In addition to these cellular components, cytokine profiles in intestinal secretions provide insight into the inflammatory environment associated with infection. In natural infection, Long et al. assessed the role of fecal cytokines in the resolution of ETEC disease. Fecal concentrations of IL-6, IL-8, IL-4, IL-5, IL-10, monocyte chemoattractant protein 1 (MCP-1), tumour necrosis factor-α (TNF-α), and interferon-γ (IFN-γ) were measured (60). They observed that elevated levels of TNF-α, IL-6 and IFN-γ were associated with increased duration of infection while CXCL-8 appeared to promote resolution (60). IL-4 was linked to shorter infection duration, while high IL-10 was not adequate in clearing infection (60). Together, these findings suggest that the balance between pro-inflammatory and regulatory cytokines may influence the clinical course of ETEC infection. However, cytokine measurements alone do not identify the cellular sources of these mediators and therefore provide only indirect insight into mucosal cellular immune responses.

Furthermore, epithelial barrier disruption during ETEC infection may permit cytokine leakage into the gut lumen, necessitating cautious interpretation of these measurements, as intestinal barrier function is known to be dynamically altered during mucosal inflammation (70).

#### 4.2.3 Innate mucosal responses

Besides the activity of various innate lymphoid cells (ILCs) and phagocytes, innate mucosal responses involve AMPs produced by specialized intestinal epithelial cells i.e., Paneth cells which constitutively express HD-5, HD-6, and lysozyme, whereas β-defensins and cathelicidins are mainly expressed by enterocytes, and hBD-1 is expressed in normal healthy small and large intestine epithelium (65).

In duodenal biopsies obtained from patients with watery diarrhea, immunomorphometry showed that Paneth cells contain significantly higher amounts of HD-5 at convalescence and in healthy controls compared to the acute stage. Similarly, levels of the AMP LL-37 were reduced during acute infection, although messenger ribonucleic acid (mRNA) expression remained unchanged (65). In. contrast, mRNA expression of hBD-2 was significantly elevated during the acute phase of watery diarrhoea compared to convalescence and healthy controls, while hBD-1, HD-5, HD-6 and lysozyme mRNA expression remained stable (65). Together, these findings suggest that ETEC infection is associated with dynamic regulation of mucosal antimicrobial peptides, reflecting activation of innate defence mechanisms at the intestinal surface.

Lactoferrin, a glycoprotein found in mucosal secretions, especially breast milk is another of the innate immune frontline defences against enteric pathogens such as ETEC especially for breastfeeding infants (69). In a vaccine study, Qadri et al. measured lactoferrin concentration in fecal samples to ensure that vaccine-induced SIgA responses measured during a vaccine trial were not due to breast milk contamination (69). This highlights the importance of accounting for innate mucosal factors when interpreting antibody measurements in young children.

#### 4.2.4 Additional observations

The transient nature of mucosal antibody responses, particularly secretory IgA, suggests that antibody concentrations decline relatively rapidly following infection or vaccination (27). However, repeated exposure and booster immunization are thought to promote immunological memory and may enable more robust responses upon subsequent encounters with ETEC antigens (27). Svennerholm et al. also observed comparable anti -LPS response frequencies in plasma and stool (68), highlighting the close interplay between systemic and mucosal compartments.

Despite the central role of mucosal immunity in protection against ETEC, direct assessment of mucosal responses remains technically challenging and is therefore performed less frequently than systemic immune measurements. Consequently, surrogate markers and compartment-specific assays are often used to infer mucosal immune activity. However, the precise correlates of protective mucosal immunity remain incompletely defined.

**Figure 1:**
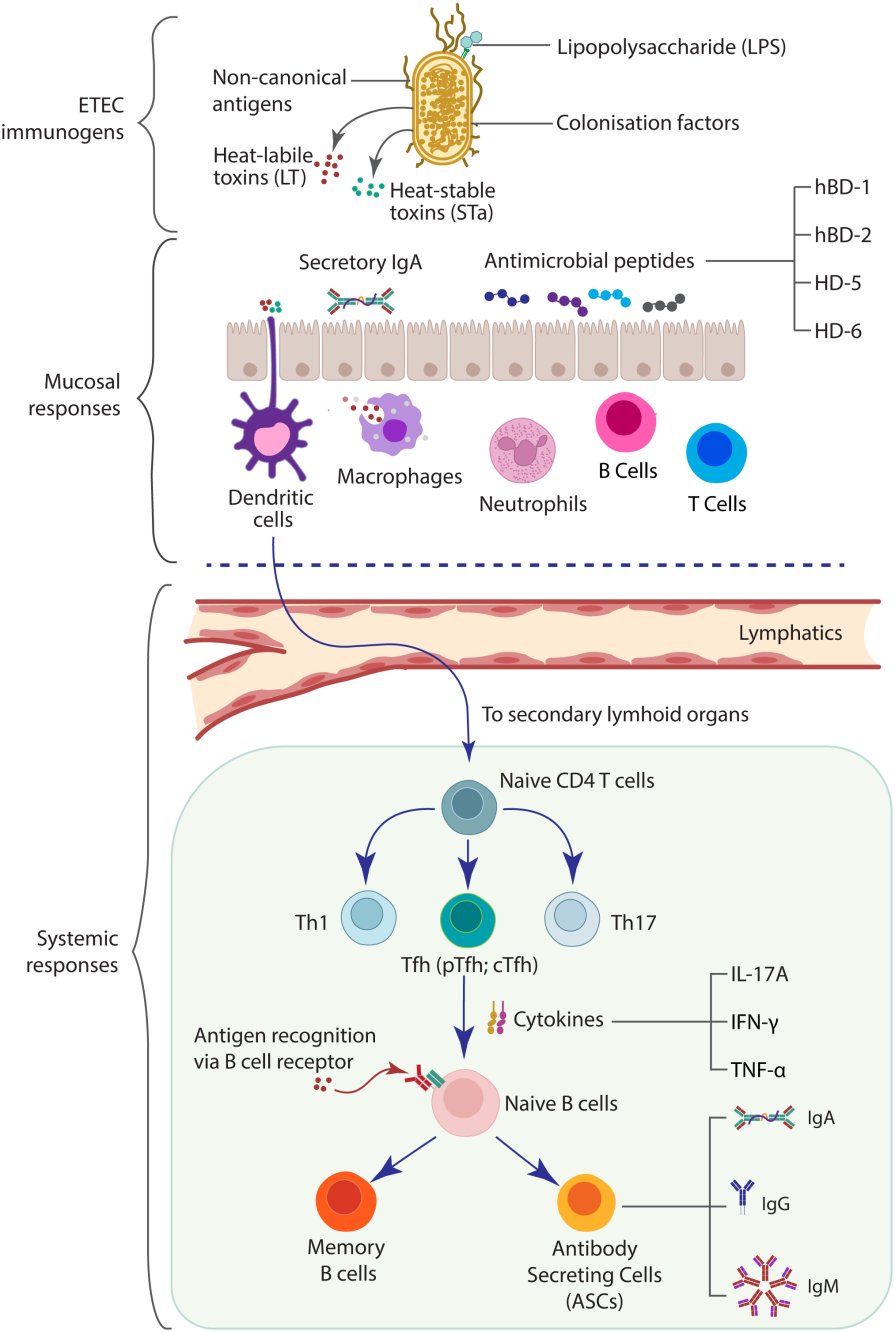
Overview of host immune responses to enterotoxigenic Escherichia coli (ETEC). The figure illustrates the host immune responses elicited following exposure to ETEC immunogens, including colonization factors (CFs), toxins, lipopolysaccharide (LPS), and non-canonical antigens (e.g., EatA, EtpA, and YghJ). At the intestinal mucosa, secretory IgA (SIgA), antimicrobial peptides (human β-defensins hBD-1 and hBD-2, and α-defensins HD-5 and HD-6), together with innate immune cells (dendritic cells, macrophages, and neutrophils) and resident B and T cells, contribute to early host defence by limiting bacterial colonization and toxin activity. Antigen presentation by dendritic cells initiates adaptive immune responses through lymphatic trafficking to secondary lymphoid organs, where naïve CD4^+^ T cells differentiate into Th1, Th17, and T follicular helper (Tfh) cells. Cytokines produced by helper T-cell subsets (IL-17A, IFN-γ, and TNF-α) promote B-cell activation and differentiation into memory B cells and antibody-secreting cells (ASCs), resulting in the production of antigen-specific IgA, IgG, and IgM antibodies that contribute to protective immunity.

**Figure 1** below summarizes the coordinated immune pathways activated during ETEC infection, showing how antigen-specific responses operate across compartments to achieve protection.

## 5 Correlates of protection

Immune correlates of protection (CoP) are immune responses, or components thereof, that are significantly and consistently associated with protection (71). Identifying a CoP against ETEC is essential for the effective design and evaluation of vaccines. However, defining a definitive CoP remains challenging due to the complex and multifactorial nature of ETEC pathogenesis, its antigenic diversity, and variability in host immune responses by age, prior exposure, and study design (e.g., challenge vs. natural infection).

Data on potential CoPs can be drawn from controlled human infection models (CHIMs), vaccine trials, and natural infection studies. These highlight systemic and mucosal responses particularly to CFs and LT, as possible correlates of protection, although a singular definitive marker is lacking.

### 5.1 Antitoxin and anti-CF antibodies

Among humoral responses, systemic IgA and IgG responses against LT and CFs are among the most extensively studied immune markers and have been repeatedly associated with protection. Early challenge studies showed that higher baseline anti-CFA/I serum IgA levels were associated with reduced risk of moderate-to-severe diarrhea (36, 38). In African children, a greater than fourfold rise in anti-LT IgA and IgG following toxoid-based vaccination correlated with robust ASC responses and reduced diarrheal incidence (38). Bhuiyan et al. further found that β7^+^ IgA- and IgG-secreting ASC frequencies to LT and CF antigens were significantly higher among protected individuals during natural infection (32).

Anti-LT neutralizing antibody responses have long been recognised as a potential correlate of protection. Early challenge and observational studies reported that individuals with higher baseline or post-infection neutralizing titers experienced reduced diarrheal risk (36, 40). Although not yet validated as a standalone correlate, toxin neutralization remains a valuable functional readout that complements binding assays and supports the role of antitoxin immunity in ETEC protection.

### 5.2 Mucosal SIgA responses

As the primary defence at the intestinal surface, mucosal secretory IgA (SIgA) is regarded as one of the most consistently associated markers of protection. Elevated fecal and duodenal SIgA levels targeting CFs or LT have been frequently associated with reduced disease severity. Qadri et al. showed that higher fecal SIgA levels in vaccinated or naturally infected individuals correlated with lower symptom burden (30). In vaccinees, Åhrén et al. demonstrated that CFA/I-specific IgA in both lavage and stool significantly correlated with protection (43).

Wennerås et al. reported that duodenal ASC responses (predominantly IgA) in vaccinees were comparable to those in naturally infected individuals (34).

### 5.3 Antibody avidity

In addition to magnitude, the functional quality of antibodies, particularly avidity, may influence protection. Antibody avidity reflects the overall binding strength of polyclonal antibodies to their antigen and can serve as a marker of immune maturation (72). A few studies have applied avidity assays in the ETEC context, showing that infection or vaccination may elicit rises in IgG and IgA avidity against LT and CFs (33, 73). Although not as widely studied as antibody titers, avidity could provide complementary insights into the durability and effectiveness of responses, especially in differentiating short-lived from long-lived protective immunity.

### 5.4 Cellular and memory responses

Among systemic cellular responses, follicular helper T (Tfh) cells and antigen-specific memory B cells provide important mechanistic insight into long-term mucosal protection.

McArthur et al. showed that non-susceptible volunteers (i.e., those who did not develop moderate-severe diarrhea) in a challenge study had higher frequencies of gut-homing pTfh cells and ETEC-specific memory B cells. These correlated with stronger mucosal IgA and bone marrow responses (59).

Cárdeno et al. later demonstrated that vaccine responders had a higher proportion of activated cTfh cells (CXCR5^+^ ICOS^+^ β7^+^), and the magnitude of their response post-vaccination predicted IgA memory after boosting, suggesting their potential as early biomarkers of vaccine efficacy (61).

### 5.5 Composite markers and practical considerations

Rather than a single marker, most studies support a composite model integrating humoral, mucosal, and cellular readouts. For example, Leach et al. observed that ALS-derived ASC kinetics differed between primary and booster vaccination and tracked closely with both serum and fecal antibody levels (44).

Novel multiplex and microarray platforms further demonstrate that protective immunity likely reflects cumulative responses to canonical (CFs, LT, ST) and non-canonical antigens (EatA, EtpA, YghJ) (20, 74).

Despite these insights, defining reliable CoPs against ETEC remains difficult due to heterogeneity in assay methodologies, antigen panels, and study populations, which complicates direct comparisons and generalizability. Host-specific factors such as age, prior exposure, nutritional status, and microbiota further complicate generalizability.

A major gap is the limited understanding of the contribution of innate immune markers (e.g., cytokines, antimicrobial peptides) and Fc-mediated antibody functions to protection against ETEC. Similarly, most studies focus on responses to LT and CFs, while the protective contribution of immunity to non-canonical antigens remains incompletely understood.

## 6 Methodological variations/assay challenges

Accurate quantification of immune responses to ETEC infection or vaccination relies on a diverse range of immunological assays targeting systemic, mucosal, and cellular compartments. These assays differ in sample type, target biomarker, detection platform, and technical complexity, all of which influence the interpretation and comparability of findings across studies. Although a diverse array of methods has been applied to study ETEC immunity, identifying reliable correlates of protection remains challenging because the immune responses that best reflect protective secretory IgA responses in the gut are not yet fully understood.

### 6.1 Serological assays

Enzyme-linked immunosorbent assays (ELISAs) remain the most widely used approach for quantifying antigen-specific IgG, IgA, and IgM in serum, plasma, and mucosal samples (stool, lavage, saliva, or breast milk) with widespread application in both challenge and vaccine studies (27, 30, 75, 76). While relatively simple and easy to standardize, results are influenced by differences in antigen preparation, antibody isotype/subclass detected, and cutoff definitions. Assay sensitivity may be affected by antigen purity and background signal. Despite these limitations, ELISAs remain the benchmark against which many newer serological platforms are compared.

Multiplex platforms, such as the Meso Scale Discovery 8-plex assay developed by Chakraborty et al, use the same principle as ELISA but allow simultaneous measurement of responses to multiple ETEC antigens with high sensitivity and inter-assay precision, correlating well with standard ELISAs but at a higher setup cost (75). These platforms correlate well with conventional ELISAs while providing improved throughput and reduced sample volume requirements, albeit at a higher setup cost. Electrochemiluminescence (ECL) assays, including the MSD, offer improved dynamic range and lower sample volume requirements but require specialized equipment (77). Thus, multiplex and ECL platforms provide advantages for large-scale studies and high-dimensional analyses but may be less accessible in resource-limited settings.

Toxin-neutralizing assays measure antibodies that block toxin activity in cell culture. They enable a more direct assessment of the protective capacity of toxin specific antibodies (35, 41, 78). Unlike binding assays such as ELISA and multiplex platforms, neutralization assays assess antibody function rather than magnitude alone. While these assays provide direct evidence of functional antibody capacity, they are technically demanding, require careful standardization, and are less commonly used in large-scale field trials compared to ELISA or multiplex approaches. Consequently, most studies rely on binding assays, with neutralization assays serving as complementary functional readouts.

### 6.2 Cellular assays

Cell-mediated immunity to ETEC is less commonly assessed but remains a critical element of immunity. Flow cytometry enables phenotyping of subsets of B and T cells, including T follicular helper (Tfh) cells and regulatory T cells, and the determination of intracellular cytokines. In addition, flow cytometric proliferation assays following antigenic stimulation provide an assessment of memory recall responses. These assays require viable PBMCs, standardized protocols, and considerable technical proficiency (59, 61). Despite their complexity, flow cytometric approaches provide high-dimensional information on cellular phenotypes and functions that cannot be captured by serological assays alone.

Cytokine quantification may also be done using ELISA, ECL platforms (e.g., MSD), or multiplex bead assays such as Luminex (50, 51, 56, 57). Compared with conventional ELISA, multiplex platforms enable simultaneous measurement of multiple analytes and permit broader characterization of immune responses. Luminex has been used in ETEC studies to quantify a wide range of cytokines, providing insights into cytokines associated with infection and vaccination.

### 6.3 Mucosal assays

The measurement of mucosal immunity is important in understanding the immune response at the site of infection. Several assays employing various techniques have been used to measure mucosal immune responses to ETEC, as shown in **Table 1**. As previously stated, mucosal IgA has been reported in fecal extracts, intestinal secretions, and gut lavage using ELISA or dot-blot microfiltration (66).

**Table 1:** Summary of assay platforms used in ETEC immunology studies.

Compartment	Assay platform	Purpose	Strengths	Limitations
Systemic	ELISA (serum)	Quantify antigen-specific IgG/IgA	Sensitive, widely available, relatively low cost	Variable antigen prep, cutoffs; time-consuming for multiple antigens
	Multiplex ELISA, Meso scale discovery	Multi-antigen antibody quantification	High throughput, small sample volume, correlates with ELISA	High initial cost, requires specialist plates
	ECL assay	IgG/IgA, cytokines	High sensitivity, broad dynamic range, low volume	Specialized equipment
Mucosal	ALS	Surrogate marker of mucosal ASC activity	Can freeze samples, ELISA compatible, field-feasible	Requires PBMC isolation, not antigen site-specific
	ASC (ELISPOT)	Enumerate antigen-specific plasma blasts	High sensitivity	Fresh PBMCs required, labour-intensive
	WGLF	Direct gut mucosal antibody measurement	Standardised, non-invasive, correlates with immunity	Requires participant prep, lab setup
	Faecal IgA ELISA/Dot-blot	Effector mucosal antibody	Non-invasive, easy collection	Variable concentration, stability issues
	Intestinal lavage	Localised mucosal antibodies	High specificity for site	Time-consuming, low feasibility for large studies
	Breast milk GM1 ELISA	Maternal antibody transfer	Non-invasive, relevant for infant protection	Only applicable to lactating women
Systemic & Mucosal	*Flow cytometry	Phenotyping, cytokine detection	Multiparametric, detects T/B cell activation	High cost, fresh samples required
	*Protein microarray	Broad antigen profiling	Identifies novel targets, multi-antigen	High technical demand, lab-limited

*These assays can be used for the measurement of both systemic and mucosal responses.

Direct sampling techniques, such as whole gut lavage fluid (WGLF), facilitate standardized, non-invasive antibody measurement throughout the intestinal tract and have been shown to have superior sensitivity for detecting mucosal IgA compared to fecal samples or blood tests (79). They are, however, dependent on participants’ cooperation, laborious, and not suitable for large-scale field studies (27).

Similarly, fecal sampling provides a less invasive alternative but is affected by variability in sample concentration, while proteases present in intestinal secretions may compromise assay reproducibility (79).

A limited number of studies have used biopsy-based methods to measure the expression of antimicrobial peptides (e.g., HD-5, hBD-2) and immune cell infiltration in the gut during ETEC infection. Although informative, these methods are invasive and therefore are rarely used outside controlled research settings (65).

Because of the practical challenges associated with direct mucosal sampling, antibody in lymphocyte supernatant (ALS) and antibody-secreting cell (ASC) assays are commonly used as surrogate markers of mucosal immunity. These assays detect transient plasmablast responses following antigen stimulation and have been shown to correlate with mucosal IgA responses measured in intestinal secretions and feces (30, 43, 80).

Enzyme-linked immunospot (ELISPOT) and ALS assays quantify IgA and IgG-producing ASCs from PBMCs following short-term *in vitro* stimulation. Compared with ELISPOT, ALS permits frozen storage of the test samples (supernatants) and subsequent ELISA-based testing, making it more convenient for use in field studies and resource-poor settings (30, 81). Both ALS and ASC are sensitive, although ALS has, in some studies, been shown to correlate better with mucosal IgA response than ASC (79). Combining these approaches with the assessment of gut-homing markers such as α4β7 or CCR9 may provide additional insight into the intestinal trafficking potential of antigen-specific cells and improve the interpretation of peripheral surrogate markers of mucosal immunity.

Longer culture-based ELISPOT assays are used to measure antigen-specific memory B cells (33). Thus, the choice of assay often represents a trade-off between sensitivity, invasiveness, feasibility, and the specific immune compartment of interest.

### 6.4 High-throughput and discovery platforms

Proteomic and immunoproteomic methods, such as protein microarrays, enable the simultaneous assessment of reactivities to dozens of canonical (e.g., CFA/I, CS6, LT, ST) and non-canonical antigens (e.g., YghJ, EatA, EtpA, FliC) (20, 25, 74, 82). Compared with conventional serological assays that focus on a limited number of predefined antigens, these platforms permit broader characterization of immune responses and facilitate the discovery of novel antigenic targets.

Such approaches have expanded understanding of immune breadth and may inform the development of future multicomponent vaccines. However, their application remains largely restricted to specialized laboratories because of the need for dedicated equipment, technical expertise, and advanced analytical approaches.

## 7 Contextual gaps/population differences

Accurate interpretation of immune responses to ETEC infection or vaccination requires consideration of demographic, geographic, and exposure-related factors that shape host immunity. These differences influence the magnitude, quality, and specificity of immune responses, and ultimately determine how findings can be generalized across populations.

### 7.1 Strain-specific immune responses

#### 7.1.1 Colonization factors and surface antigens

Immune responses to ETEC are highly strain specific with limited cross-reactivity to heterologous strains (13, 83). Several studies show that immune responses (both systemic and mucosal), especially those targeting the colonization factors such as CFA and CS antigens, are predominantly directed against homologous antigens used in vaccination trials and challenge studies (84). This strain specificity presents a challenge for vaccine development and may contribute to incomplete cross-protection between ETEC strains.

ETEC produces one or both of two toxins, LT and ST, which differ in immunogenicity. LT is highly immunogenic and induces an anti-LT antibody response upon infection with LT-producing ETEC strains, while ST is less immunogenic (83). Consequently, toxin-specific immunity depends on the infecting strain and it’s virulence profile.

In one study, Levine et al. observed that antibodies to LT alone, may not reliably protect against ETEC diarrhoea, especially if the anti-LT antibody levels decline over time. This was because; after recovery from diarrhoea caused by the ETEC strain B7A, volunteers remained susceptible to subsequent diarrheal episodes induced by the heterologous strain E 2528-C1 (83). Given that the only antigen common to both strains was the LT and considering that all four previously exposed volunteers had initially developed subsequent rises in anti-LT antibodies after their primary infection with B7A (83). These findings suggest that protection against ETEC, particularly against heterologous strains, is unlikely to depend solely on antitoxin immunity and may require sustained antibody responses as well as broader recognition of additional bacterial antigens (83).

Beyond the toxins, the primary targets for strain-specific immunity are the CFs on the bacteria’s surface. More than 25 CFs have been identified (85, 86). Several other immunogenic non-canonical antigens have been identified. Chakraborty et al. observed that volunteers of a human experimental challenge mounted robust ALS responses to YghJ, EatA, EtpA, and FliC, which if included in vaccine formulations may broaden the immune response (20).

Other reports also show that EatA, YghJ, and EtpA are immunogenic and appear to be relatively conserved (13, 74).

ETEC infections also induce IgA and IgG responses to O-specific lipopolysaccharide (LPS) on the bacterial surface. Stoll et al. observed that naturally acquired ETEC diarrhea elicits IgA and IgG responses to LT, CFA and LPS that are detectable in gut lavage, breastmilk, saliva, and serum (27). Brussow et al. noted that antibodies to LPS O78 may cross-react with other O antigens of other *E.coli* serogroups (42).

Interestingly, while colonization factor responses are strain-specific, responses to LT, particularly the B subunit LTB, appeared to be more conserved across strains (87). Together, these observations suggest that effective vaccines may require combinations of strain-specific and conserved antigens to achieve broad protection against the antigenic diversity of ETEC.

### 7.2 Age and demographic factors

#### 7.2.1 Infants and young children

Immune responses vary significantly with age. Very young children benefit from passive maternal immunity (via placental transfer of maternal IgG and through breastfeeding), which offers transient protection against infection (76, 88, 89). Breastfeeding appears to confer protection against ETEC in infancy. In Bangladesh, exclusive breastfeeding in infancy was associated with a significant reduction in risk of severe ETEC diarrhea (88), while in Mexico, high anti-LT antibody levels in breast milk lowered the risk of symptomatic infection by 90% (76). However, as the infants get older and are weaned off, waning passive immunity and exposure through food other than breast milk increases the risk of infection, with the hazards of asymptomatic ETEC infection increasing by as high as 400% (76, 89).

By toddlerhood, repeated natural infections drive the development of active immunity, reflected in increasing anti-ETEC antibodies in serum and mucosal secretions.

Seroepidemiologic data from endemic settings show low antibody prevalence in infancy, with a rise in the second year of life.

In Ecuadorian children, low prevalence of antibodies to ETEC LPS and LT was observed in the first six months of life, followed by marked increase in 6- to 12-month-olds, to as high as 90% prevalence by the second year of life (90) Similarly, Clemens et al. found that the age-related pattern of anti CFA/I and CFA/II antibodies among controls paralleled the age-related incidence of ETEC diarrhoea among the cases, with negligible levels in infancy that rise to peak in the second year of life (91). Ryder et al. observed the highest antibody prevalence in children aged 1–5 years due to frequent exposure (89).

Similar age-dependent responses are observed in clinical trials, where older children (12–59 months) mount stronger mucosal and systemic responses to vaccine antigens than infants (6–11 months) (69, 77), suggesting that immunological maturity and previous exposure contribute to vaccine responsiveness.

#### 7.2.2 Adults

Adults in endemic areas generally have broader immunity and reduced disease severity due to exposures over time (89). However, antibody levels wane in the absence of re-exposure, and immunity in adults may rely on immune memory to infection rather than high circulating antibody levels (89).

In contrast, adults from non-endemic regions are highly susceptible. Among US travellers to Mexico, 74% of those infected with LT-ETEC seroconverted, with a fourfold increase in antibody titres between arrival and departure samples with young adult travellers being more likely to seroconvert and become ill (37).

Differences between endemic and non-endemic populations are also reflected in vaccine responses. A vaccine trial in Egypt which enrolled adults, school children and preschoolers showed an age-related response difference: after oral vaccination with the candidate ETEC vaccine, children showed higher IgA seroconversion rates to each of the ETEC antigens in the vaccine (70-96%) compared to adults (31-69%), possibly because many of the adults had baseline antibodies that made fourfold titre rises less common (92). This difference was attributed to higher baseline antibody levels in adults, which made fourfold rises in antibody titres less frequent. Together, these findings suggest that prior exposure and immunological history are major determinants of both infection outcomes and vaccine-induced immune responses.

### 7.3 Geographic variations

#### 7.3.1 Endemic and non-endemic populations

Exposure to enteric pathogens varies geographically and may impact the immune profile against ETEC (89, 90). Infections are more common in endemic regions such as LMICs (parts of South Asia, Latin America, and Africa) where repeated exposure from infancy results in high baseline immunity by adulthood (89). In rural Bangladesh and other endemic areas, most children have detectable antibodies to the major antigens (LT and CFs) by age 2-5 (88, 89).

Marked differences in antibody prevalence have been observed between populations and even between geographically proximate regions. Ryder et al. found that Panamanian children had acquired higher antibody titers by age five compared to children in the US canal zone region, showing the regional variations even within endemic areas (89). Another study comparing the prevalence of anti-ETEC antibodies in Ecuadorian and German children found a low prevalence of LT-specific IgG in German children compared to a high prevalence in Ecuadorian children (90).

These high baseline levels in adults in endemic areas contrast sharply with the near-absence of antibodies in adults from non-endemic regions. Travelers’ diarrhoea is a direct manifestation of this gap: among U.S. travellers to Mexico, up to 60% developed diarrhoea and many seroconverted during short-term stays (37). Likewise, military personnel to endemic regions show low pre-deployment titers that rise upon exposure (67).

Geographical differences are not limited to immune status but also extend to circulating ETEC strains and colonization factor profiles. Even within endemic areas, regional differences exist in dominant ETEC serotypes and colonization factors. For example, Panamanian children acquired higher CFA/I antibody titers than children from the U.S. Canal Zone region, despite geographic proximity (89). Such differences affect how vaccines perform across regions: in endemic settings, a priming dose may function as a booster in adults with pre-existing memory, whereas in naïve populations it elicits primary responses.

#### 7.3.2 Exposure history and immune memory

Cumulative exposure, whether from repeated infections or environmental contamination, affects both baseline immunity and responsiveness to re-exposure. In a human challenge study, rechallenge with the same ETEC strain protected volunteers from illness despite low measurable responses to LPS and LT in ALS and serum (93).

This finding suggests that conventional serological measurements may underestimate protective immunity and that immunological memory or pre-existing mucosal responses may contribute to protection.

Consequently, immune responses following reinfection may differ substantially according to prior exposure history. Individuals living in endemic settings may experience mild or asymptomatic reinfections accompanied by modest increases in antibody titres, whereas immunologically naïve travellers are more likely to develop symptomatic disease and mount robust acute responses.

#### 7.3.3 Host and environmental factors influencing susceptibility and immune responses

Host-related and environmental factors may also contribute to variability in susceptibility to ETEC infection and in the magnitude of immune responses. Studies have shown that host genetics, particularly histo-blood group antigens (HBGAs), influence susceptibility to symptomatic disease. Qadri et al. observed that ETEC diarrhea was more prevalent among Bangladeshi children with blood groups A and AB than among those with blood group O (94). More recently, Kumar et al. demonstrated that individuals with blood group A developed more severe diarrhea following challenge with the LT-producing strain H10407, an effect linked to interactions between the adhesin EtpA and blood group A glycans (95). Similarly, Ahmed et al. reported that Bangladeshi children with the Lewis phenotype Le(a+b−) were more susceptible to symptomatic ETEC infection, suggesting that HBGAs influence host-pathogen interactions and disease severity (96).

Nutritional status may further influence the immune response to ETEC infection and vaccination. Undernutrition and micronutrient deficiencies are common in endemic settings and have been associated with impaired mucosal immune responses (63). Dietary mycotoxin exposure, particularly to aflatoxins, is common in many endemic settings and has been linked to environmental enteric dysfunction, systemic immune activation, and immunosuppression, which may further modulate susceptibility and vaccine responsiveness (97).In addition, environmental enteric dysfunction (EED), a condition characterized by chronic intestinal inflammation and impaired barrier function, has been proposed as a contributor to the reduced immunogenicity of oral vaccines observed in children living in low-resource settings (98–100). Emerging evidence also suggests that the intestinal microbiome may influence susceptibility to infection. Higginson et al. observed distinct microbial signatures between symptomatic and asymptomatic ETEC carriers, while Sauvaitre et al. demonstrated that interactions between ETEC, the mucus layer, and the gut microbiome may play an important role in the pathogen infectious cycle (101, 102). Collectively, these observations highlight the importance of considering host and environmental factors when interpreting immune responses and evaluating vaccine performance across populations.

## 8 Discussion

Immune responses to ETEC span systemic, mucosal, humoral, and cellular compartments, reflecting the pathogen’s multi-antigenic nature and the complexity of protection. Serum IgA and IgG, mucosal IgA, and ASC responses to canonical antigens such as CFs and LT remain the most reported immune responses (30, 36, 91). More recent studies highlight circulating T follicular helper (cTfh) cells, memory B cells, and cytokines as contributors to durable immunity (59, 61, 74). Although secretory IgA is widely regarded as a key mediator of protection at the intestinal surface, the immune responses that best reflect protective mucosal immunity remain incompletely understood. In addition, the protective antigens required for broad protection have not been fully defined, reflecting the diversity of ETEC strains and the complexity of host–pathogen interactions (71).

Emerging evidence also suggests that cellular immunity contributes to the development and maintenance of protective responses against ETEC. Studies investigating pTfh and cTfh cells have demonstrated associations with mucosal IgA responses and long-term memory, while cytokine profiling indicates the involvement of both Th1- and Th17-associated pathways. Collectively, these findings suggest that ETEC infection and vaccination induce coordinated cellular responses that interact closely with humoral immunity and may contribute to durable protection. However, compared with humoral responses, cellular and innate immune mechanisms remain comparatively less well characterized.

A major barrier lies in the heterogeneity of immunoassays. Conventional ELISAs remain the mainstay for antibody quantification, but variability in antigen preparation, isotype detection, and cutoff definitions hinders comparability across studies (77). Likewise, ALS and ASC assays, which are widely used as surrogate measures of mucosal immunity, are subject to similar limitations and differ mainly in feasibility and field applicability (32). Similarly, mucosal sampling strategies, ranging from fecal extracts to whole-gut lavage, vary in sensitivity and logistical practicality (27, 79). These differences mean that studies assessing the same antigen often arrive at divergent conclusions. Without harmonized assay platforms and standardized reagents, even large datasets risk fragmentation rather than synthesis.

Population context further complicates interpretation. In endemic regions, infants are initially protected by maternal antibodies but become highly susceptible after weaning, with repeated exposures in early childhood shaping immune repertoires (76, 88). Adults in such settings often exhibit partial protection despite waning antibody titers (89), while travelers from non-endemic regions remain vulnerable to severe illness upon first exposure (37). More recently, host-related factors including histo-blood group antigens, nutritional status, environmental enteric dysfunction, and the intestinal microbiome have emerged as additional contributors to heterogeneity in disease susceptibility and vaccine responsiveness.

Geographic variability in strain distribution, CF expression, and antigen prevalence adds another layer of complexity, influencing both natural immunity and vaccine performance (86).

Pathogen diversity compounds these challenges. While CFs and LT have been central targets, their heterogeneity limits broad protection (13, 83). ST, though epidemiologically important, remains a weak immunogen, and immunity to LT alone does not guarantee cross-strain protection (83). Encouragingly, non-canonical antigens such as EatA, YghJ, and EtpA have shown promise as broadly immunogenic candidates (20, 74), suggesting that future vaccines may require multi-antigen formulations combining canonical and novel non-canonical targets to achieve global coverage.

**Figure 2:**
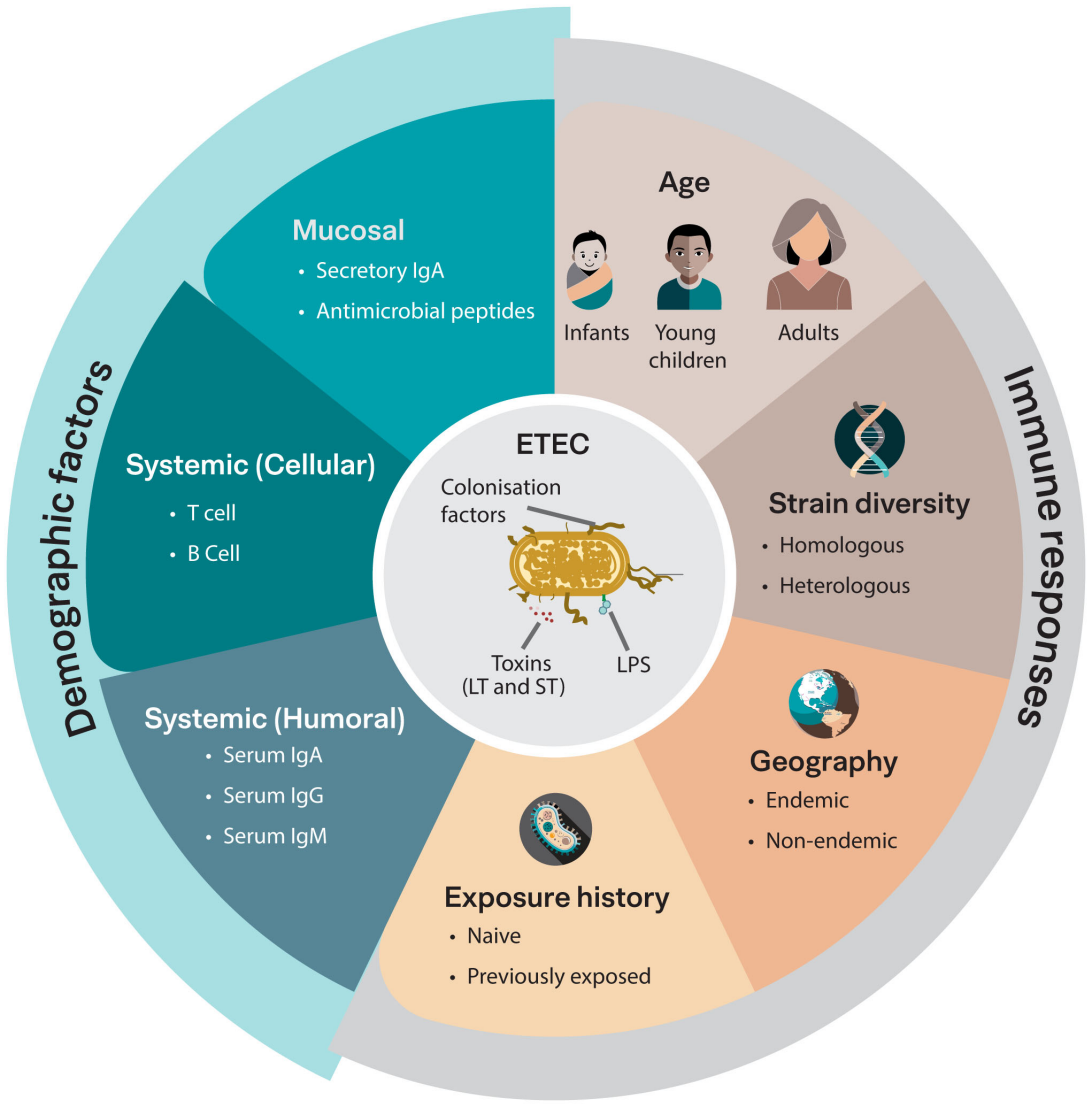
Immune responses to ETEC antigens and their functional roles in protection. Schematic representation of the major ETEC antigens and the immune responses they elicit. Colonization factors (CFs, e.g., CFA/I, CS3, CS5) mediate adherence to the intestinal epithelium, stimulating antigen-specific secretory IgA (SIgA) that blocks colonization. The heat-labile toxin (LT), particularly its B subunit (LTB), induces both systemic and mucosal antibodies that neutralize toxin activity. The heat-stable toxin (ST), though weakly immunogenic, may trigger modest antibody responses. Additional antigens including EatA, EtpA, YghJ, flagellin (FliC), and O-specific lipopolysaccharide (LPS) contribute to broader immune recognition. Mucosal SIgA acts at the intestinal surface to prevent adherence and toxin delivery, while systemic IgG provides secondary defence by neutralizing circulating antigens. Together, these immune components form a multi-layered defence, though responses vary by strain and prior host exposure.

Taken together, the evidence presented here indicates that protection against ETEC is unlikely to hinge on a single marker or antigen. Instead, a multidimensional profile integrating systemic antibody breadth, mucosal SIgA kinetics, early ASC activation, and T-cell help may provide a more accurate readout of protective immunity. This is summarized conceptually in **Figure 2**, which highlights the interplay of host age, exposure history, geography, and pathogen strain diversity in shaping the magnitude, breadth, and durability of immune responses to ETEC.

To move the field forward, four key priorities may need to be addressed. Firstly, assay harmonization by establishing standardized protocols, validated reagents, and reporting criteria for ELISA, ALS, and ASC platforms, particularly for mucosal endpoints (79, 81). Secondly, contextualization of vaccine trial designs to account for baseline immunity, prior exposure, and local strain distribution and virulence characteristics in vaccine evaluations (88, 92). Differences in enterotoxin production and pathogenic potential among circulating strains may influence disease severity and complicate the interpretation of vaccine-induced responses. High baseline antibody titers may obscure changes in vaccine-induced antibody titers, complicating interpretation of seroconversion endpoints. Thirdly, conducting integrated immune profiling to incorporate systems immunology approaches that combine humoral, cellular, and mucosal readouts to define composite CoPs (20, 61, 82, 103). Emerging technologies such as systems vaccinology, machine learning approaches for biomarker discovery, and high-dimensional mucosal immune profiling may further facilitate the identification of predictive signatures of protection and accelerate vaccine development. Fourthly, improving our understanding of host-related factors, including genetics, nutritional status, environmental enteric dysfunction, and the gut microbiome, and their influence on immune responses and vaccine performance.

By aligning methodological rigor with a nuanced understanding of population and pathogen variability, the field can accelerate the identification of actionable correlates of protection. This will be critical for advancing ETEC vaccine development from proof-of-concept to global deployment.
